# The role of health literacy in vaccination behaviours: a systematic review: Claudia Isonne

**DOI:** 10.1093/eurpub/ckac131.350

**Published:** 2022-10-25

**Authors:** LM Siena, C Isonne, A Sciurti, MR De Blasiis, G Migliara, C Marzuillo, C De Vito, P Villari, V Baccolini

**Affiliations:** Public Health and Infectious Diseases, Sapienza University, Rome, Italy

## Abstract

**Background:**

Health literacy (HL) is recognized as a driver of health-promoting behaviors, including preventive actions. However the influence of HL on vaccines uptake remains unclear. This study aimed at summarize the evidence on the role of HL in vaccination behaviors.

**Methods:**

PubMed, Scopus, and Web of Science were searched. Observational studies of any design conducted worldwide, published through June 2021 and investigated the association between HL and vaccination intention or status using HL validated tools were included. Any vaccine was considered. An adapted Newcastle-Ottawa Scale was used to assess quality.

**Results:**

Twenty-one articles were included, 6 investigated intention to vaccinate and 15 explored vaccination status. Studies of the first group had a cross-sectional design, considered anti-COVID-19 vaccination and were judged of high or fair quality. Population investigated was heterogeneous as well as the tool used to assess HL. Five analysis provided adjusted estimates. HL seemed not influence the vaccination intention in 3 studies whereas adequate HL was associated with positive attitude to get vaccinated in the remained 3 ones. The majority of articles assessed vaccine status, had a cross sectional design (N = 11) and were of high quality (N = 8). The HL tool more frequently used was TOFHLA (N = 5), sample investigated was heterogeneous included parents of children who received vaccinations (N = 5). Four articles considered multiple vaccinations, thus providing a total of 19 analysis. Vaccine against influenza was the most investigated (N = 11) and 15 studies provided adjusted estimates. No association was found between HL and the receipt of vaccination in 11 analysis, whereas HL levels significantly influenced vaccination uptake in 8 studies.

**Conclusions:**

Health literacy did not seem to strongly influence people decision on vaccinations. Difference in population and vaccines considered, but also in tool used to measure HL might explain the heterogeneity of the results.

**Key messages:**

• The impact of HL on vaccination behaviours remains controversial.

• Efforts to extend the studies on targeted populations applying a comprehensive HL measurement tool should be devised.

